# Tumor-specific migration routes of xenotransplanted human glioblastoma cells in mouse brain

**DOI:** 10.1038/s41598-023-51063-7

**Published:** 2024-01-09

**Authors:** Rajesh Kumar Gupta, Mia Niklasson, Tobias Bergström, Anna Segerman, Christer Betsholtz, Bengt Westermark

**Affiliations:** 1 Deparment of Immunology, Genetics and Pathology, Rudbeck Laboratory, Uppsala University, Uppsala, Sweden; 2 Department of Medical Sciences, Cancer Pharmacology and Computational Medicine, Uppsala University, Uppsala, Sweden; 3 Department of Medicine-Huddinge, Karolinska Institutet Flemingsberg Campus, Huddinge, Sweden

**Keywords:** Cancer, Cell biology, Molecular biology, Oncology

## Abstract

The migration of neural progenitor cells (NPCs) to their final destination during development follows well-defined pathways, such as along blood vessels. Cells originating from the highly malignant tumor glioblastoma (GBM) seem to exploit similar routes for infiltrating the brain parenchyma. In this report, we have examined the migration of GBM cells using three-dimensional high-resolution confocal microscopy in brain tumors derived from eight different human GBM cell lines xenografted into immunodeficient mice. The primary invasion routes identified were long-distance migration along white matter tracts and local migration along blood vessels. We found that GBM cells in the majority of tumors (6 out of 8) did not exhibit association with blood vessels. These tumors, derived from low lamin A/C expressing GBM cells, were comparatively highly diffusive and invasive. Conversely, in 2 out of 8 tumors, we noted perivascular invasion and displacement of astrocyte end-feet. These tumors exhibited less diffusive migration, grew as solid tumors, and were distinguished by elevated expression of lamin A/C. We conclude that the migration pattern of glioblastoma is distinctly tumor cell-specific. Furthermore, the ability to invade the confined spaces within white matter tracts may necessitate low expression of lamin A/C, contributing to increased nuclear plasticity. This study highlights the role of GBM heterogeneity in driving the aggressive growth of glioblastoma.

## Introduction

Highly specialized blood vessels in the brain function not only as supply routes that allow selective passage of molecules to the brain parenchyma but also play an important role in guiding migratory cells to their final destination^1^. Vessels support migratory cells in several ways including by providing guidance cues, to act as physical scaffolds, and promote cell survival and proliferation. Blood vessels have been shown to guide migration of subventricular zone (SVZ)-derived neural progenitor cells (NPCs) to reach their final destination both in physiological and pathological conditions^2–6^.

Malignant glioblastoma (GBM) is the most common and lethal brain tumor in adults. A contributing factor to the poor prognosis is that surgical resection is unachievable because of the extensive and long-range invasion of tumor cells into the surrounding brain tissue. Like normal NPCs, glioblastoma cells have been shown to use blood vessels to invade brain parenchyma. Vessel-associated perivascular invasion is considered to constitute one of the major routes of diffuse infiltrative growth of GBM^7–15^. Perivascularly invading glioma cells not only proliferate and migrate along blood vessels^8^ but are also reported to displace astrocyte end-feet and disrupt the blood–brain barrier^14^.

Although extensive perivascular invasion is reported in many experimental models^8,14^, there are GBM models that do not show perivascular invasion. Most glioma stem cell (GSC)-derived tumors in rodents do not show prevalent perivascular invasion but are highly diffusive and invasive with a preference of migration along white matter tracts^16–20^. In the present study, we have conducted a high-resolution microscopic analysis of the migratory routes of molecularly and clinically annotated human glioblastoma-derived GSCs^20^ (hgcc.se) after xenografting to the striatum of immunodeficient NOD SCID mice. We were able to define two major patterns of migration: (i) local migration with or without vessel-association and disruption of astrocyte end-feet, and (ii) long-distance migration along white matter tract without vessel-association.

## Results

### Xenotransplanted glioblastoma cells migrate along two main routes in a cell line specific manner

**Figure 1:**
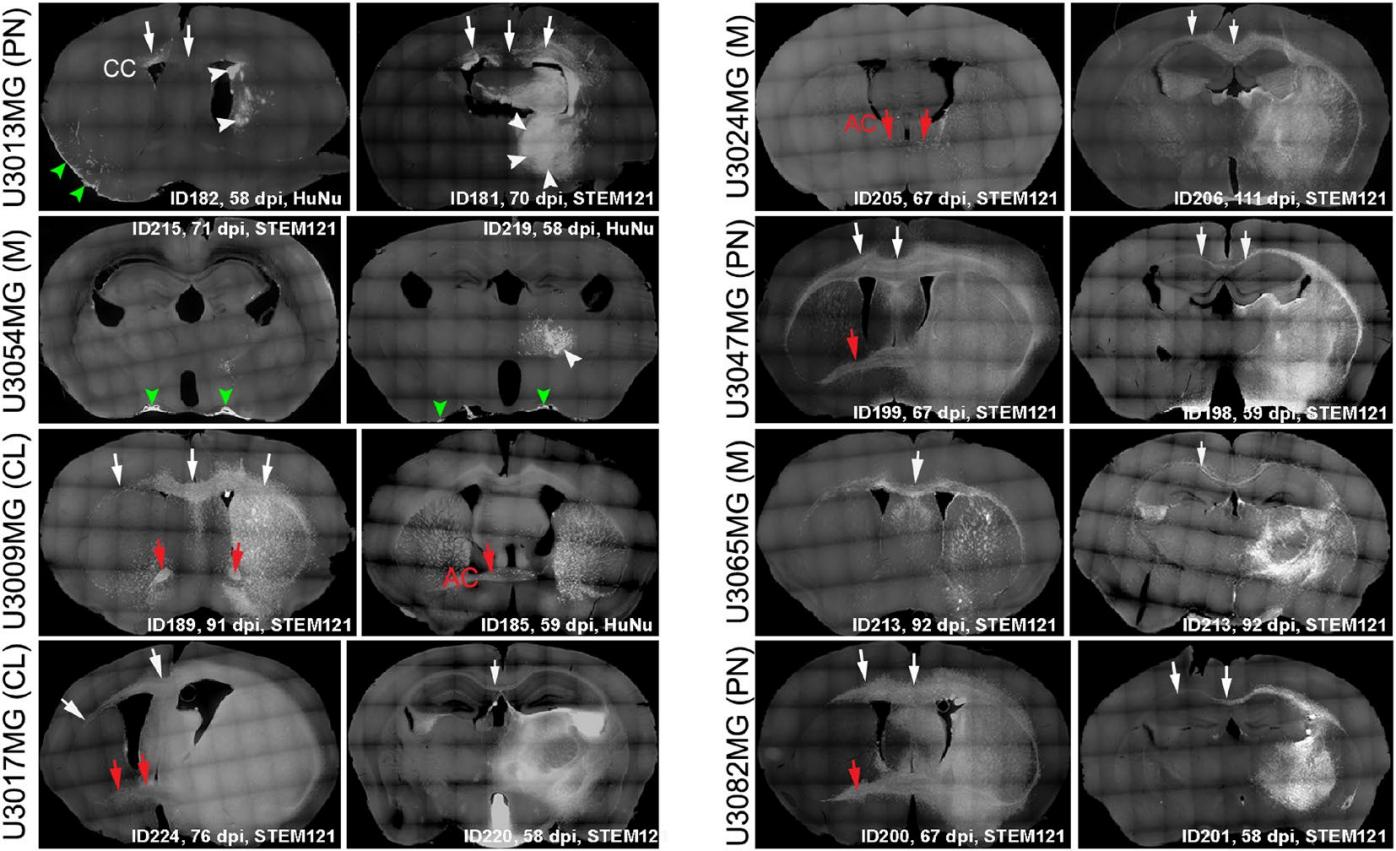
In vivo growth patterns of tumors derived from eight GSC lines. Whole coronal sections stained with STEM121 or HuNu antibodies to localize human glioblastoma cells, followed by imaging using the Leica SP8 microscope tile scan function. Animal ID and tumor age in days post injection (dpi) are indicated. *CC* corpus callosum (white arrows), *AC* anterior commissure (red arrows); Subarachnoid space (green arrowheads); Solid tumor mass (white arrowheads). GBM molecular subtypes of GSC lines are indicated: *CL* classical, *M* mesenchymal, *PN* proneural.

To study the migration routes of human glioblastoma cells in mice, eight human GSC lines—representing three major GBM subtypes (classical, mesenchymal, and proneural)^20^—were orthotopically injected into NOD SCID mice (5 mice per cell line, Supplementary Table 1). Tumor-bearing brains were sectioned and stained with STEM121 (human cytoplasm) or HuNu (human nuclei) antibodies to visualize human glioma cells, followed by analysis of tumor growth pattern and invasion. All tumors exhibited invasive growth to varying extent and with different growth patterns, and we could not observe a specific tumor growth type for the individual GBM subtypes (Fig. 1). Most of them showed extensive invasion, particularly along white matter tracts. For these tumors, we regularly observed migration along corpus callosum or anterior commissure into the contralateral hemisphere. U3013MG and U3054MG did not migrate efficiently through the corpus callosum but still formed locally invasive tumors (Fig. 1). In addition, we found that glioma cells from these tumors accumulated in the subarachnoid space (Fig. 1 and Supplementary Fig. 1a). These cells appeared to invade the perivascular compartment of vessels penetrating the brain from the subarachnoid space (Supplementary Fig. 1b and Supplementary Movie 1). Other tumors did not migrate into the subarachnoid space in spite of being often located in close proximity to pia mater (Fig. 1 and Supplementary Fig. 1a). We next examined to what extent the different invasive patterns were associated with migration along the vasculature.

### Association of glioma cells with vasculature is not a consistent feature of xenotransplanted glioblastoma cell line

**Figure 2:**
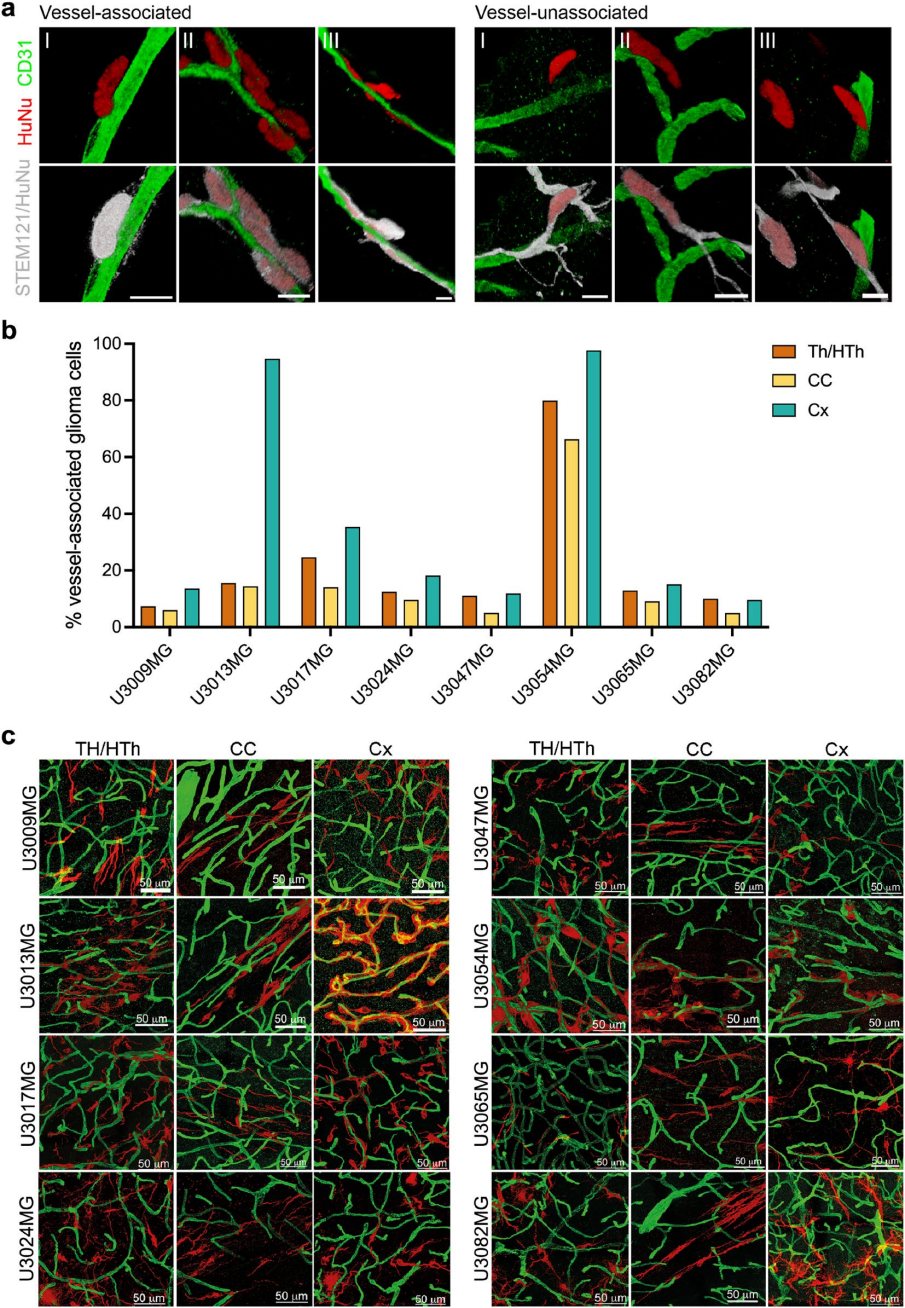
Association of tumor cells with vasculature at the invasive front in different brain regions. (**a**) Regions with different types of blood vessel-associated (left) and vessel-unassociated (right) glioma cells. Coronal sections were sequentially stained with HuNu (red/white) and STEM121 (white) antibodies to visualize human glioma cell nucleus and cytoplasm, respectively. Blood vessels (green) were visualized by anti-CD31 antibody staining. Each image represents a snapshot of a 3D image. Scale bar, 10 µm. (**b**) Bar graph showing blood vessel-associated glioma cells in different brain regions, thalamus/hypothalamus (Th/HTh), corpus callosum (CC), cortex (Cx). For details, see Supplementary Table 2 and Supplementary Fig. 2. (**c**) Immunofluorescence stainings of thalamus/hypothalamus (Th/HTh), corpus callosum (CC), cortex (Cx) regions using STEM121 antibodies against human glioma cells (red) and CD31 antibodies against blood vessels (green) on coronal sections. Each image represents a snapshot of a 3D image and is supported by a movie (see Supplementary Movies 2–25).

To investigate the association between blood vessels and migrating tumor cells at the invasive front of the tumors, coronal sections were stained with STEM121/HuNu and anti-CD31 antibodies to visualize human tumor cells and blood vessels, respectively. We imaged large areas of the invasive front at high-resolution and quantified vessel-associated tumor cells in three different regions—corpus callosum (CC), cortex (Cx) and thalamus (Th)/hypothalamus (HTh) (Fig. 2, Supplementary Fig. 2, Supplementary Table 2, and Supplementary Movies 2–25). Both position of nuclei and cytoplasmic processes were considered (Fig. 2a; see also “Experimental procedure”). Notably, the locally invasive U3054MG and U3013MG tumors demonstrated a high degree of tumor cell association with blood vessels (Fig. 2b,c). U3054MG tumor cells exhibited a substantial level of vessel-association in all three regions (≈ 80% in Th/HTh, ≈ 66% in CC, and ≈ 97% in Cx), while U3013MG tumor cells showed a very high level of vessel-association in Cx (approximately 94%) but not in Th/HTh (15.5%) or CC (14.4%). In contrast, the other cell lines showed an overall low level of association with blood vessels, ranging from 5 to 35% (Fig. 2b,c). Next, we examined the association between tumor cells and blood vessels in detail.

### Disruption of astrocyte end-feet but intact pericyte coverage in high-level vessel-associated tumors

**Figure 3:**
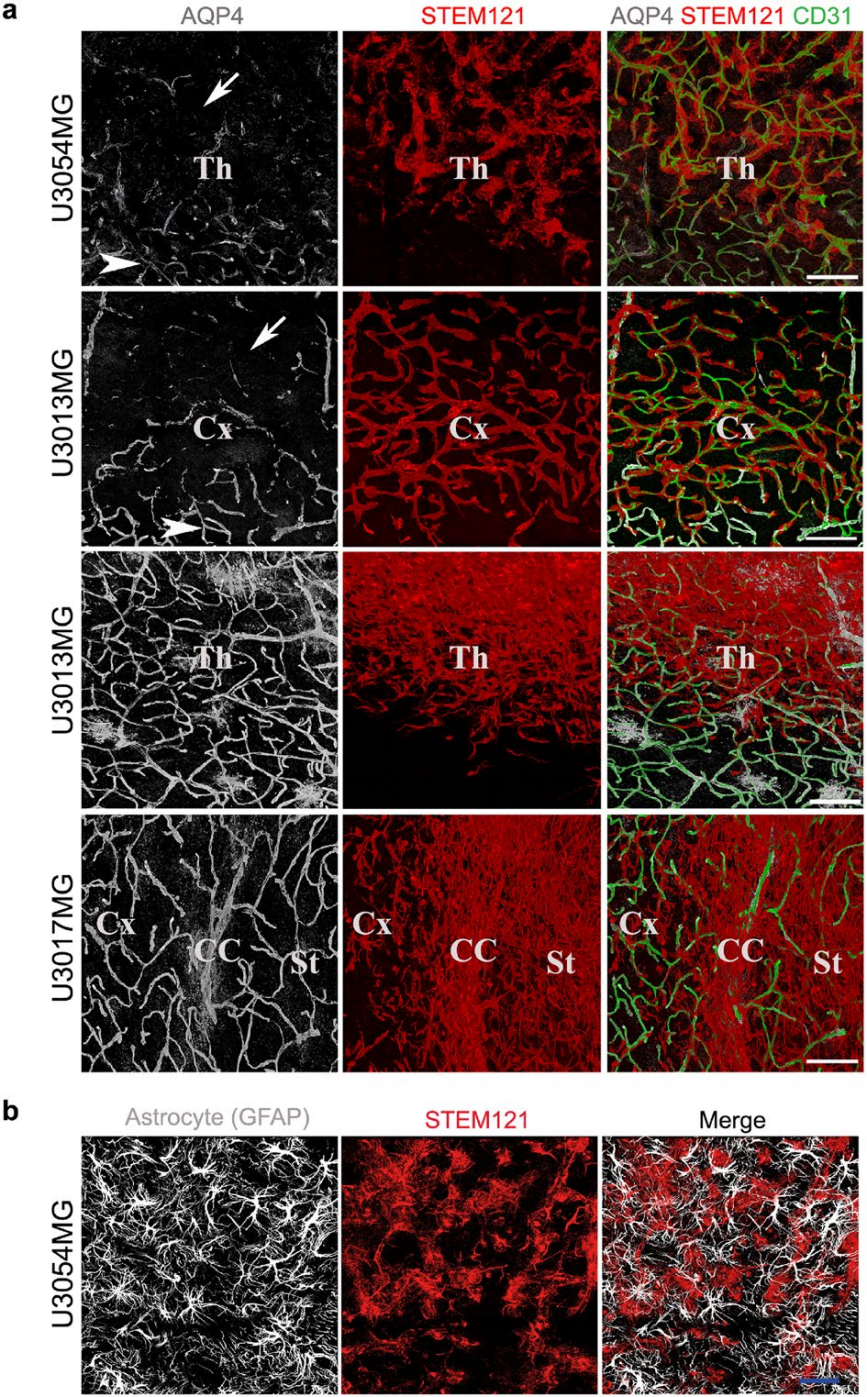
(**a**) Loss of astrocyte end-feet in regions of high-level vessel-associated glioma cells. Immunofluorescent staining of human glioma cells (STEM121, red), astrocyte end-feet (AQP4, white), and blood vessels (CD31, green) on coronal brain sections. z-stacks were taken by Leica confocal microscope using 63 × objective in tile scan mode to cover a large area. Each image represents a snapshot of a 3D image. Arrow indicates the glioma cell occupied region with loss of astrocyte end-feet and arrowheads indicate regions with visibly no loss of astrocyte end-feet from vessels. *Cx* cortex, *Th* thalamus, *CC* corpus callosum, *St* striatum. Scale bar: 100 µm. For astrocyte end-feet analysis data on all tumors, see Supplementary Fig. 3. (**b**) U3054MG tumor section (thalamus) stained with GFAP and STEM121 antibodies. Scale bar, 100 µm.

**Figure 4:**
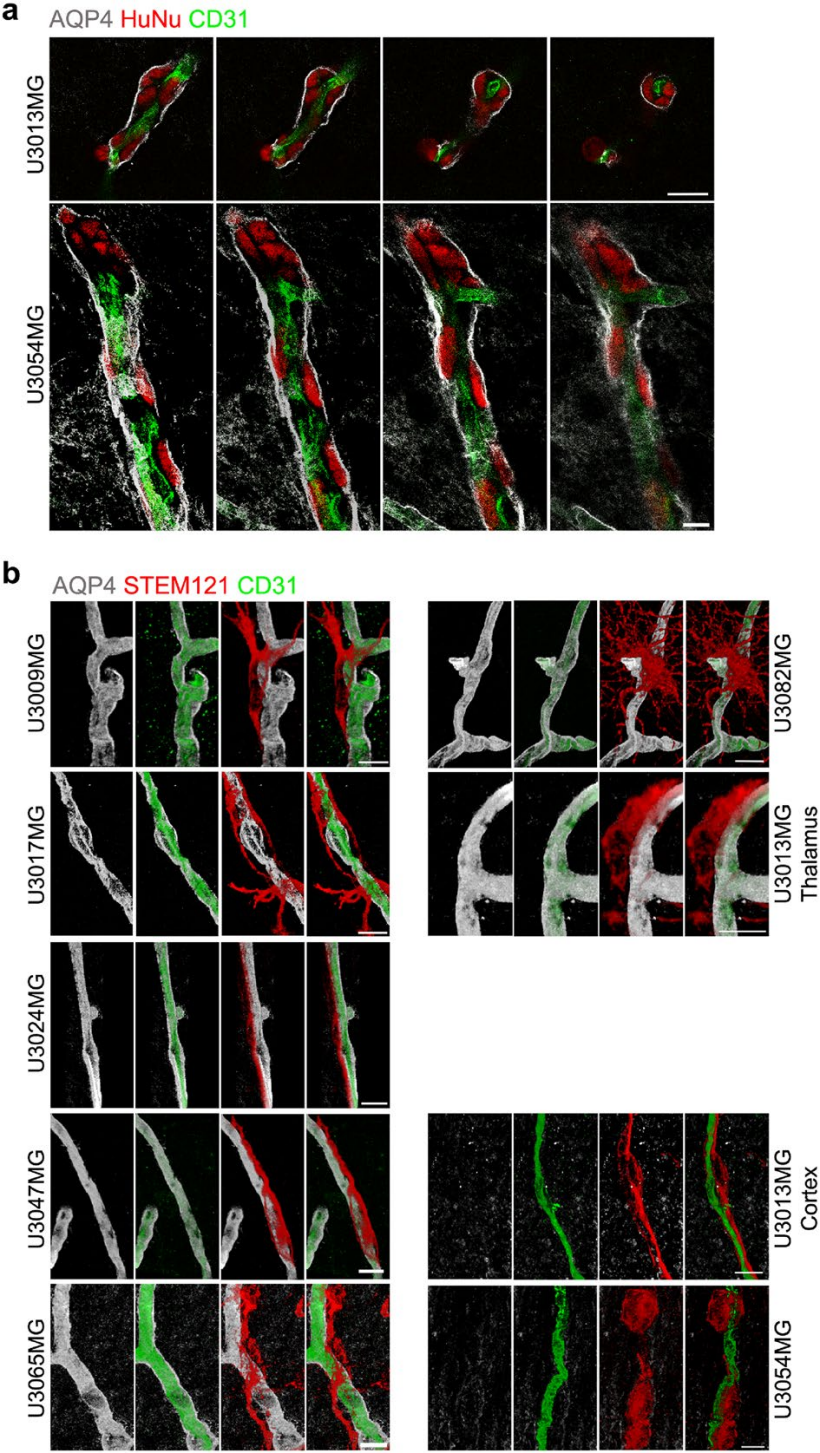
Perivascular invasion and displacement of astrocyte end-feet by glioma cells. (**a**) U3013MG and U3054MG glioma cells in perivascular space (between blood vessel and astrocyte end-feet). Coronal sections were stained with HuNu (red, human glioma cells), AQP4 (white, astrocyte end-feet) and CD31 (green, blood vessels) antibodies and z-stacks of selected regions were taken by Leica confocal microscope using 63 × objective. Scale bar, 20 µm. (**b**) Interaction of human glioma cells (STEM121, red) with astrocyte end-feet and blood vessels. Each image represents a snapshot of a 3D image. Scale bar, 10 µm.

Previous studies have shown that glioma cells that are associated with blood vessels tend to migrate in the perivascular space—the area between astrocyte end-feet and endothelial cells—and thereby cause a local disruption of the blood brain barrier^14^. To investigate whether this was the case in our settings, we stained for astrocyte end-feet and endothelial cells using anti-aquaporin 4 (AQP4) and anti-CD31 antibodies, respectively, along with STEM121 or HuNu to visualize the human glioma cells. Loss of astrocyte end-feet was indeed observed in tumor regions with high tumor cell–vessel association, such as in U3054MG and the cortical region of U3013MG (Fig. 3a and Supplementary Fig. 3), despite ample presence of astrocytes in the tumor tissue (Fig. 3b). Glioma cells from these tumors were seen to invade perivascular regions (Fig. 4a), indicating that the presence of invading tumor cells caused retraction of the end-feet, as reported by others^14^. However, we did not detect any change in CD13 (a pericyte marker encoded by the *Anpep* gene) staining in these regions, suggesting that pericyte coverage was largely unaffected (Supplementary Fig. 4). Unlike U3013MG (cortex) and U3054MG (all regions), tumor cells from all other tumors did not invade the perivascular spaces. The tumor cells in close contact to vessels in these tumors were found on the parenchymal side of the astrocyte end-feet (Fig. 4b).

### U3013MG and U3054MG tumors may use different routes to enter the perivascular space

**Figure 5:**
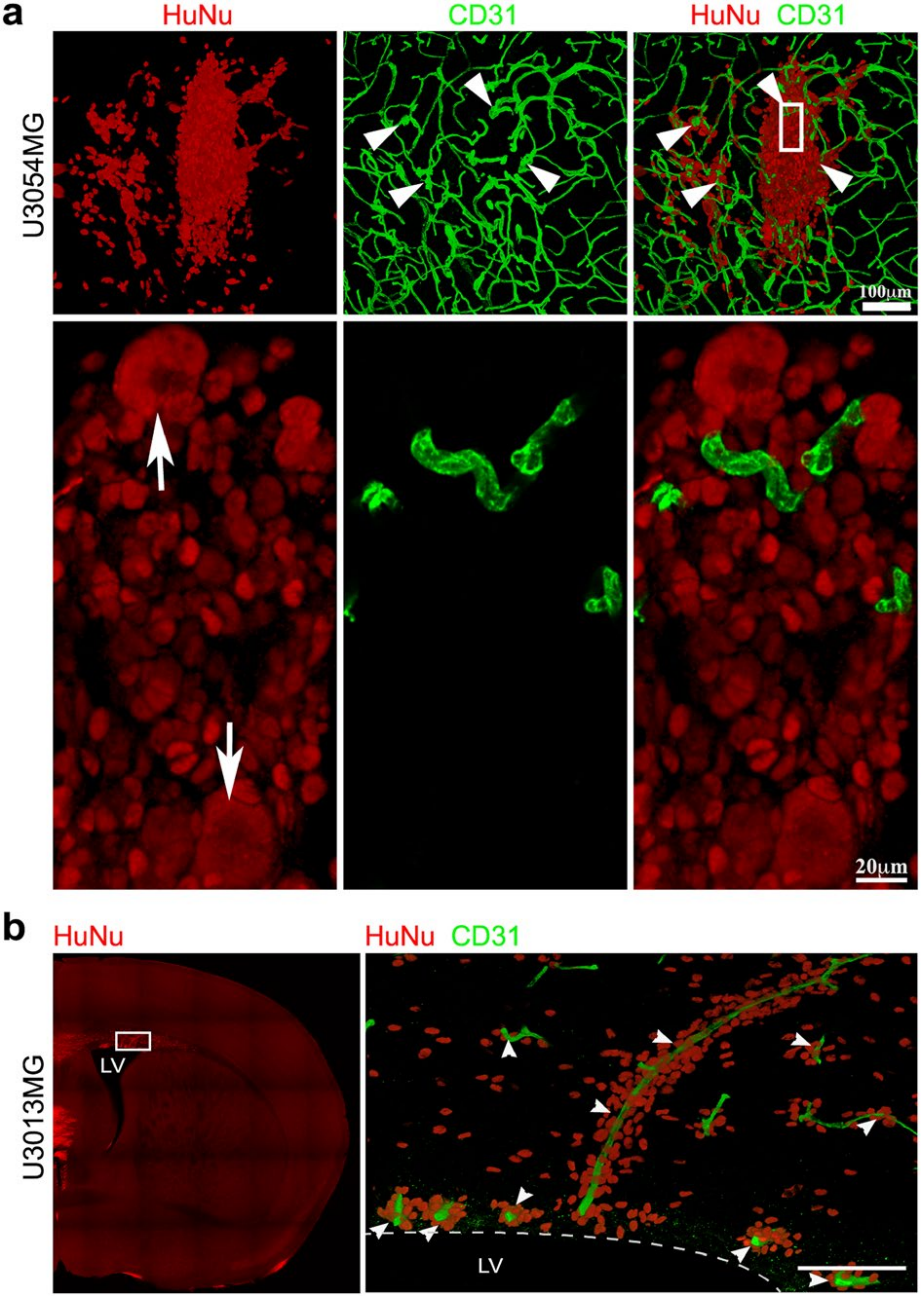
U3013MG and U3054MG tumors may use different routes to enter the perivascular space. (**a**,**b**) Immunofluorescent staining of coronal sections using HuNu (red, human glioma cells) and CD31 (green, blood vessels) antibodies. (**a**) U3054MG tumors possess features like compact growth, excessive nuclear atypia and abnormal dilated vessels (arrowheads). The upper panel shows tumor growth at the site of glioma cell transplantation (3D image, all z-stacks). The lower panel (zoom of rectangle in upper panel, few z-stack 3D image) shows nuclear morphology (giant nuclei, arrows) and compactness of nuclei at the center of the tumor. z-stacks were taken by Leica confocal microscope tile scan mode, 63 × objective. (**b**) Lateral ventricle-associated U3013MG tumor cells have a high tendency to associate with blood vessels. The left panel shows the part of whole coronal section that was imaged. The right panel shows the 3D image of z-stacks of the selected area (marked by a rectangle in the left panel image) taken by Leica confocal microscope using 63 × objective in tile scan mode. Arrowheads indicate blood vessels-associated glioma cells. Scale bar, 100 µm. *LV* lateral ventricle.

Vessel co-option ability refers to the ability of tumor cells to utilize pre-existing blood vessels in the surrounding tissue for their own nutrient supply^10^. U3054MG tumor cells demonstrated the ability to closely associate with blood vessels (Fig. 2), and in particular large- and medium-sized blood vessels were often encased by tumor cells, implying vessel co-option ability (Fig. 5a). Moreover, we observed clusters of tumor cells closely associated with vessels, referred to as secondary clusters^8^, in specific locations. These areas of compact tumor tissue were characterized by abnormal, dilated vessels and highly pleomorphic tumor cell nuclei (nuclear atypia) (Fig. 5a).

As mentioned above, U3013MG cells showed frequent association with vessels only in the cortex, but not in regions around the primary inoculation site in the striatum (Fig. 2b). This led us to speculate that U3013MG cells may not have the intrinsic ability to actively invade perivascular spaces. We carefully examined the cortical zone near corpus callosum and found that cells at the invasive front in close proximity to corpus callosum, presumably moving from corpus callosum to cortex, did not show extensive vessel-association (Supplementary Fig. 5). On the contrary, in cortical regions adjacent to the lateral ventricles, U3013MG cells were highly associated with blood vessels (Fig. 5b). Displacement of astrocyte end-feet was prominent in this region, and presumably these tumor cells may have entered the perivascular spaces via the lateral ventricles. As previously mentioned, cells also seemed to have the ability to migrate from the subarachnoid space into the perivascular space of cortical vessels (Supplementary Fig. 1b).

### Differential expression of lamin A/C suggests that the invasion pattern of glioblastoma cells is connected to nuclear plasticity

**Figure 6:**
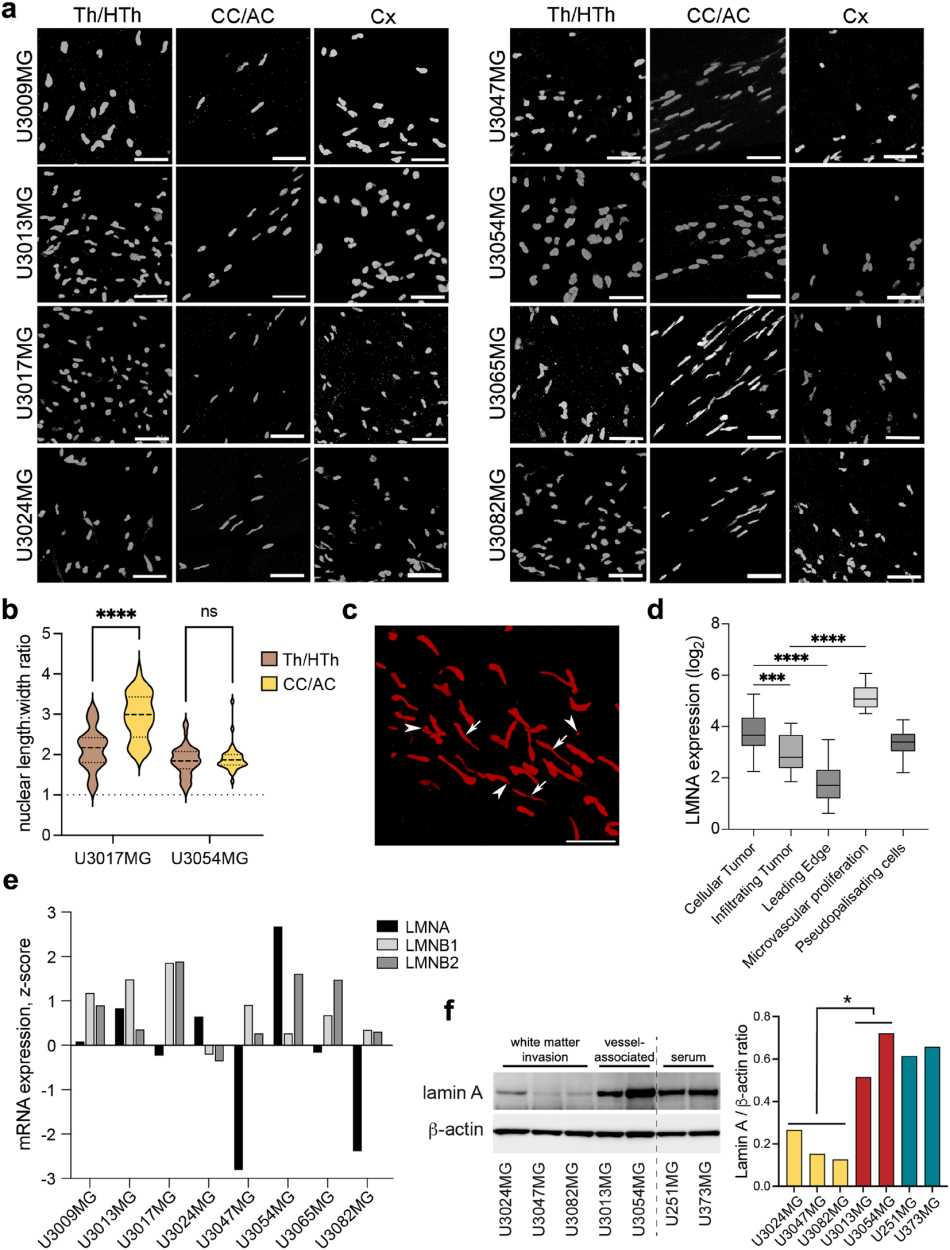
Invasion pattern of GBM cells in relation to nuclear shape and lamin A/C expression. (**a**) Nuclear shape of tumor cells in different brain regions. 100 µm thick coronal sections were stained with HuNu antibody to visualize human glioma cell nucleus. Selected regions were scanned using tile scan function by Leica confocal microscope. Each image represents a snapshot of 3D image of selected adjacent z-stacks. Scale bar, 50 µm. *CC* corpus callosum, *AC* anterior commissure, *Cx* cortex, *Th/HTh* thalamus/hypothalamus. (**b**) Violin plot showing analysis of nuclear shapes (length:width ratio, spherical = 1) in invasive front regions of corpus callosum/anterior commissure (CC/AC) and thalamus/hypothalamus (Th/HTh) in U3017MG (white matter-associated) and U3054MG (vessel-associated) tumors. ****p < 0.001, 2-way ANOVA. (**c**) Nuclear plasticity and fragmentation (micronuclei) in U3065MG tumor cells migrating in the corpus callosum. The image represents a snapshot of a 3D image. Elongated nuclei and nuclear fragmentation/micronuclei are marked by arrows and arrowheads respectively. Scale bar: 50 µm. See related movie—Supplementary Movie 26. (**d**) LMNA expression in different tumor cell populations from the Ivy Glioblastoma Atlas (Gliovis). ***p < 0.001; ****p < 0.0001, unpaired *t*-tests. (**e**) Expression (z-scores) of lamin genes—LMNA (Lamin A/C), LMNB1 and LMNB2 (lamin B)—in the GSC lines, obtained from the Human Glioblastoma Cell Culture (HGCC) database (hgcc.se) and plotted as bar graph. (**f**) Western blot analysis of Lamin A protein levels in five GSC lines exhibiting preferential white matter migration (U3024MG, U3047MG and U3082MG) and vessel-associated migration (U3013MG and U3054MG), as well as in two classical serum-grown GBM cell lines. Quantification of Lamin A band intensity (normalized to β-actin band intensity) is shown to the right (*p = 0.019, unpaired *t*-test).

Our results indicate that glioblastoma cells are able to associate with the vasculature through two distinct pathways, one by direct vessel co-option (U3054MG) and one indirectly via the subarachnoid space/lateral ventricles. However, the majority of the cell lines (6 out of 8) did not use the vascular invasive route, but instead invaded the white matter and migrated along the corpus callosum and anterior commissure to reach the contralateral hemisphere. To accomplish this, tumor cells have to squeeze through tight spaces between the nerve fibers in the white matter. Consequently, the tumor cells migrating through corpus callosum were extremely elongated (Supplementary Fig. 6a) and displayed oblong and distorted nuclei (Fig. 6a–c). Nuclei of white matter-associated tumors (e.g., U3017MG) are significantly elongated in cells migrating in CC/AC, as compared to cells invading Th/HTh, whereas no difference was observed in perivascularly-associated tumors (e.g., U3054MG) (Fig. 6b). Moreover, nuclear fragmentation was commonly observed in the migrating cells, suggesting physical damage caused by shearing forces while traveling through narrow spaces within the brain parenchyma (Fig. 6c and Supplementary Movie 26). These observations contrasted with the nuclei of tumor cells located in thalamus and cortex, which were spheroid or elliptical in shape (Fig. 6a,b).

The nucleus, being the largest and stiffest organelle in the cell, requires a plastic and deformable nuclear matrix for the cell to penetrate narrow spaces. This nuclear stiffness is largely regulated by lamin A, a type of intermediate filament that constitutes the nuclear matrix^21^. Therefore, we hypothesized that the different invasion patterns observed in the GSC lines might be due to their individual lamin A content. To investigate this, we first analyzed the lamin A mRNA expression levels in cells isolated from different tumor parts, including invading cells, using the Ivy Glioblastoma Atlas (IvyGAP)^22^ in the GlioVis data portal^23^. We found that the lowest expression of lamin A mRNA was observed in the leading edge, i.e., in invading cells (Fig. 6d). To further explore this relationship, we analyzed the relative levels of lamin mRNA (LMNA, LMNB1, LMNB2) expression in HGCC lines^20^ in relation to their invasive routes. All invasive white matter associated tumors, except for U3024MG cells, expressed very low lamin A/C mRNA (Fig. 6e). This correlated with their lack of perivascular invasion (Fig. 4b) and high tendency to migrate along white matter tracts (Fig. 1). In contrast, U3054MG cells expressed high levels of lamin A/C, whereas U3013MG cells expressed moderate levels (Fig. 6e). To confirm this finding on the protein level, we analyzed three cell lines with preferred white matter invasion (U3024MG, U3047MG and U3082MG) along with the vessel-associated cell lines U3013MG and U3054MG. We also included two classical serum-cultured cell lines, U251MG and U373MG, both of which form bulk tumors and do not migrate through corpus callosum^18^. As shown in Fig. 6f, the protein data matched the mRNA expression, with significantly higher levels of lamin A expressed in U3054MG and U3013MG cells compared to the lines exhibiting white matter invasion. The classical serum-cultured cell lines also showed high lamin A expression (Fig. 6f). To summarize, our results indicate that the nuclear stiffness of glioblastoma cells play a role for their invasive route preferences when they migrate through the brain parenchyma.

## Discussion

The long-range invasion of migrating glioblastoma cells makes radical surgical resection impossible. The present study highlights the main routes of glioblastoma invasion, namely along the vasculature and myelinated tracts. Given the architecture of the brain vasculature, it is difficult to envision how perivascular migration can cause rapid and efficient distant invasion in the brain. Rather, it is reasonable to assume that while short-distance invasion into the juxtatumoral brain parenchyma may be facilitated by the vasculature, distant migration occurs mainly along white matter tracts. The clinical implication of this distinction is obvious. Tumor recurrences occur almost invariably locally, i.e., close to the operation field. Remaining relapse-generating cells may thus hide within the peritumoral vasculature and be the founder of the glioblastoma recurrence. Cells migrating through corpus callosum may be of less clinical importance in current treatment of glioblastoma. These cells cannot be removed, but their relapse potential may be delayed and revealed only after more radical surgery, such as hemisphere-ectomy^24^.

A striking result from our study is that the migration pattern of a given glioblastoma cell line is specific to that cell line and reproducible between experiments. Thus, migration pattern is dictated by intrinsic, glioma cell autonomous mechanisms. A literature search suggests that GBM growth patterns of xenotransplantation models largely depend on the method by which implantation material is derived. It is well known that human GBM cell lines established and maintained in serum-supplemented media often grow as demarcated tumor lumps, and, when invading, they chose a perivascular invasion pattern^8,10,25^. Similarly, human GBM tumors maintained in the flanks of mice also prefer perivascular invasion pattern when transplanted in the rodent brain^14,26^. In contrast, glioblastoma lines grown in serum-free neural stem cell medium form highly diffuse infiltrative tumors in rodents^18^, as did the majority of cell lines in the present study. The reason behind the difference in invasion pattern between cell lines derived and maintained using different methodologies is not known, but it is likely to be explained by a combination of selection and serum-induced phenotypic change. Neural stem cell medium favors growth of stem-cell-like tumor cells, which resemble normal neural stem cells in their migratory behavior. It is well known that the addition of serum to glioblastoma culture leads to dramatic changes in phenotype often described in terms of differentiation^27^, which may explain their inability to migrate along myelinated tracts^28^.

Glioblastoma cells migrating in corpus callosum were extremely elongated with thin, oblong nuclei. These observations align with previous findings that propose varied brain structures exert a significant influence on the shape of glioma cells^7^. In order to migrate through white matter tracts, the cell must be flexible enough to be able to squeeze in the narrow space between the myelinated fibers. Since the nucleus is larger and stiffer than any other cytoplasmic or cellular organelles, the cell´s ability to migrate through narrow spaces is limited by the stiffness of the nucleus. In turn, nuclear stiffness is affected by the content of lamin A/C. High level of lamin A/C results in a stiff nucleus, which may contribute to a low ability to penetrate surrounding tissue. Knock-down of lamin A/C yields a plastic nucleus and thereby increases cell migration through narrow pores, however at the expense of nuclear stability, with a resulting increased risk for fragmentation^21^. We found that white matter associated invasive tumor cells had low expression of lamin A/C but expressed lamin B1/B2^20^. Moreover, the thin, migrating cells often contained nuclear fragments (Fig. 6 c), most likely as a consequence of shearing forces applied to the nucleus during the cell’s migration in the white matter, in line with experimental data^21^. Like cell lines cultured in serum, U3054MG and U3013MG tumors expressed comparatively high levels of lamin A/C. These cells tended to form localized solid tumor masses, displaying limited migration through white matter tracts. Additional investigations are imperative to elucidate the specific role of lamin A/C in glioblastoma invasion, along with for instance analyses of the release of enzymes responsible for degrading extracellular matrix components.

Vessel co-option ability of U3054 tumor cells apparently provided a route for local spread through perivascular spaces near the transplantation site. These tumors showed features of classical serum grown cell lines including enlarged blood vessels and highly pleomorphic nuclei. Interestingly, U3054MG cells were derived from a recurring tumor. Glioblastoma recurrences are known to be phenotypically different from primary tumors. Whether glioblastoma recurrences in general present with a less invasive phenotype than the primary tumors is an interesting subject for future investigation.

U3013MG tumors were unique both in terms of migratory pattern and ability to acquire perivascular invasion. Although these tumors were not as invasive as white matter associated tumors, tumor cells were often found migrating in the parenchyma in small groups. In addition, when these tumors acquired perivascular invasion, presumably via the lateral ventricles, they were as diffusive as white matter associated tumors, supporting the idea that perivascular space is indeed a potential route of tumor cell invasion. The presumed lateral ventricle-mediated perivascular invasion appears to be associated with accumulation of cells in lateral ventricles but how this leads to perivascular invasion is not clear and needs further research.

The present study did not reveal any relationship between glioblastoma subtype and migration pattern. Considerably larger studies, preferably including paired primary and recurrent tumors, are required to answer this question. A more burning issue is to unravel the molecular mechanisms that determine the migratory routes. Since our study shows that the mode of migration is tumor specific, comparative transcriptomics and proteomics and other profiling studies on a large number of cell lines with defined migration patterns are likely to be informative.

## Materials and methods

### Cell culture

All eight glioma cell lines (U3009MG, U3013MG, U3017MG, U3024MG, U3047MG, U3054MG, U3065MG, and U3082MG) were obtained from the HGCC collection^20^ (hgcc.se) and maintained in culture according to HGCC guidelines. Briefly, cells were grown on laminin (Cat. No. L2020, Sigma Aldrich) coated tissue culture dishes (Primaria culture dish, Cat. No. 353802, Corning) in serum-free culture medium (1:1 ratio of Neurobasal Medium (Cat. No. 21103-049, Thermo Fisher) and DMEM/F-12, GlutaMAX™ (Cat. No. 10565-018, Thermo Fisher)), supplemented with 10 ng/ml FGF-2 (Cat. No. 100-18B, Peprotech), 10 ng/ml rhEGF (Cat. No. AF-100-15, Peprotech), N-2 (Cat. No. 17502048, Thermo Fisher) and B-27 solution (Cat. No. 17504044, Thermo Fisher). U251MG and U373MG cells were obtained from our local cell repository and were cultured on Falcon standard tissue culture dishes (Fisher Scientific) in DMEM-GlutaMAX™ media, 1% penicillin/streptomycin and 10% heat-inactivated fetal bovine serum (Thermo Fisher).

### Animals and intracranial transplantation of GCS lines

All animal experiments were performed in accordance with institutional guidelines and approved by Uppsala Animal Ethics Committee. The study is reported in accordance with ARRIVE guidelines. Seven to eight weeks old female NOD SCID mice (NOD/MrkBomTac-*Prkdc*^*scid*^, Taconic) were used for the analysis and they were maintained in the Uppsala University EBC animal facility under standard conditions throughout the experiment. Mice were transplanted with glioma cells to the right striatum. Briefly, 2 µl of cell suspension containing 50,000 cells/µl were injected at stereotactic coordinates 1.5 mm mediolateral and 0.0 mm anteroposterior relative to bregma at a depth of 3.0 mm. Body weight and animal health was monitored weekly and animals were euthanised by carbon dioxide, and brain was collected at fixed time points or when any sign of sickness was observed (Supplementary Table 1).

### Vibratome sectioning and immunohistochemistry

Animal was perfused with PBS followed by 4% paraformaldehyde (PFA). Brain was dissected and treated with 4% PFA for 4 h at 4 °C. Brain was stored in PBS containing 0.02% Azide at 4 °C until sectioned by vibratome. Immunostaining was performed on 100 µm thick coronal sections of brain. Briefly, sections were treated with blocking/permeabilization buffer (1% BSA, 0.5% Triton X 100 in PBS) for 1 h at room temperature followed by primary antibody treatment. After a brief washing with antibodies dilution buffer (0.5% BSA, 0.25% Triton X 100 in PBS), sections were treated with secondary antibodies followed by washing and mounting using ProLong™ Gold Antifade Mountant (Cat. No. P36930, Thermofisher). Stained sections were analyzed by Leica confocal microscope. Often a large area using tile scan mode in confocal microscope was scanned which was trimmed to select region of interest. Primary antibodies—Mouse HuNu antibody (Cat. No. MAB1281, Millipore), Mouse STEM121 antibody (Cat. No. Y40410, TaKaRa Clontech), Goat Anti-CD31 antibody (Cat. No. AF3628, R&D systems), Rabbit Anti-AQP4 antibody (Cat. No. AB2218, Millipore) were used in our study. The following secondary antibodies were used in this study: Donkey Anti-Goat IgG Alexa 488 (Cat. No. A-11055, Thermofisher), Donkey Anti-mouse IgG Alexa 555 (Cat. No. ab150110, AbCam), Donkey Anti-mouse IgG Alexa 647 (Cat. No. A-31571, Thermofisher), Donkey Anti-Rabbit IgG Alexa 647 (Cat. No. A-31573, Thermofisher).

### Counting of vessel-associated glioma cells

100 µM thick coronal sections were sequentially stained first with HuNu/Anti-mouse IgG Alexa 555 antibodies combination followed by STEM121/Anti-mouse IgG Alexa 647 antibodies combination as described above. Since both secondary antibodies were anti-mouse, nuclei of tumor cells in this setting stained with both Alexa 555 (Red) and Alexa 647 (white) whereas cytoplasm stained only with Alexa 647 (white). Large areas close to invasive front were scanned using 63 × objective of confocal microscope and stitched 3D images were used to count vessel associated glioma cells manually using Leica LAS AF software. Criteria for considering a glioma cell vessel associated/unassociated are shown in Fig. 2a. Both position of nucleus and cytoplasmic processes were considered for evaluating a cell for vessel association. Tumor cells in close association (usually less that 3 μm) with blood vessels were considered vessel-associated (Fig. 2a, left panel), except in some situations (Fig. 2a, right panel). Migrating cells that cross the blood vessel (judged by cytoplasmic processes of the migrating cells) were not considered vessel-associated even if the cell nucleus was very close to the vessel. See Supplementary Table 2 for quantification details.

### Quantification of nuclear shape

Nuclei lengths and widths were measured (arbitrary unit) manually in 3D images. The length/width ratio was calculated (1 = spherical) from 25 to 50 cells/section. Nuclei from three HuNu stained sections per region were measured (U3017MG-Th, 3 mice; U3017MG-CC/AC, 2 mice; U3054MG-Th, 2 mice; U3054MG-CC/AC, 2 mice). Calculated width:length ratios were plotted as a violin graph using Graph Pad prism.

### Western blot analysis

Cells were scraped and lysed in 1× RIPA lysis buffer (10XRIPA, Roche) with protease inhibitors (cOmplete, Merck Life Science) and phosphatase inhibitors (PhosStop, Merck Life Science), 30 min on ice, followed by 13,000 rpm centrifugation and collection of supernatant. Protein concentration was measured using the Pierce BCA protein assay kit (Pierce, Rockford, USA) followed by absorbance readings using a CLARIOstar microplate reader (BMG Labtech). Proteins were separated on a 4–12% Bis–Tris polyacrylamide gel in MOPS buffer (NuPAGE, Thermo Fisher) and transferred to a nitrocellulose membrane using the Power Blotter System (NuPAGE). The filter was blocked in 5% BSA/TBS-T, incubated with primary antibody (polyclonal Lamin A (PA5-81211, Invitrogen); monoclonal beta-actin (A5441, Sigma Aldrich)) in 1% BSA/TBS-T overnight, 4 °C, followed by washing and incubation with secondary HRP-coupled antibodies (anti-rabbit and -mouse, GE Healthcare), RT, 1 h. ECL Select Reagent (Cytiva) and Amersham Imager 680 (Cytiva) were used for protein detection. Quantification of western blot band intensity was performed in Adobe Photoshop 2024 using 82 px × 32 px rectangles. Background intensity adjacent to each individual band was subtracted. Each sample’s Lamin A band intensity was normalized to its β-actin band intensity. Statistical analysis was performed using unpaired *t*-test in GraphPad Prism, version 10.1.1.

### Statistical analysis

Unpaired *t*-tests and 2-way ANOVA analyses were performed using GraphPad Prism, version 10.1.1.

### Supplementary Information

Supplementary Information.

Supplementary Movie 1.

Supplementary Movie 2.

Supplementary Movie 3.

Supplementary Movie 4.

Supplementary Movie 5.

Supplementary Movie 6.

Supplementary Movie 7.

Supplementary Movie 8.

Supplementary Movie 9.

Supplementary Movie 10.

Supplementary Movie 11.

Supplementary Movie 12.

Supplementary Movie 13.

Supplementary Movie 14.

Supplementary Movie 15.

Supplementary Movie 16.

Supplementary Movie 17.

Supplementary Movie 18.

Supplementary Movie 19.

Supplementary Movie 20.

Supplementary Movie 21.

Supplementary Movie 22.

Supplementary Movie 23.

Supplementary Movie 24.

Supplementary Movie 25.

Supplementary Movie 26.

## Data Availability

The datasets used and/or analyzed during the current study are available from the corresponding author on request.

## Abbreviations

AC: Anterior commissure
BSA: Bovine serum albumin
CC: Corpus callosum
CL: Classical GBM
Cx: Cortex
GBM: Glioblastoma multiforme
GSC: Glioma stem cell
HTh: Hypothalamus
M: Mesenchymal GBM
NOD SCID: Nonobese diabetic severe combined immune deficiency
NPCs: Neural precursor cells
PBS: Phosphate-buffered saline
PFA: Paraformaldehyde
PN: Proneural GBM
SVZ: Subventricular zone
Th: Thalamus
