# Supplementary material for: Tumor-specific migration routes of xenotransplanted human glioblastoma cells in mouse brain
Source: Sci Rep. 2024 Jan 9;14:864. doi: 10.1038/s41598-023-51063-7 (PMC10776844; doi:10.1038/s41598-023-51063-7)
Supplement: Supplementary file 1 — Supplementary Information. [file 41598_2023_51063_MOESM1_ESM.docx]

**Supplementary Tables and Figures**

**Supplementary Table 1**

| **Cell line** | **Animal ID** | **Survival time** | **Tumor size** |
| --- | --- | --- | --- |
| **U3009MG**  Classical  Passage 17 | 185 | 59 days | Small |
|  | 186 | 128 days | No tumor |
|  | 187 | Died during surgery | NA |
|  | 188 | 74 days | Small |
|  | 189 | 91 days | Intermediate |
| **U3013MG**  Proneural  Passage 12 | 180 | 85 days | Large |
|  | 181 | 70 days | Intermediate |
|  | 182 | 58 days | Small |
|  | 183 | 70 days | Small |
|  | 184 | 70 days | Intermediate |
| **U3017MG**  Classical  Passage 9 | 220 | 58 days | Intermediate |
|  | 221 | 71 days | Large |
|  | 222 | 73 days | Large |
|  | 223 | 76 days | Large |
|  | 224 | 76 days | Large |
| **U3024MG**  Mesenchymal  Passage 9 | 205 | 67 days | Small |
|  | 206 | 111 days | Intermediate |
|  | 207 | 69 days | Small |
|  | 208 | 59 days | Small |
|  | 209 | 22 days | Very small |
| **U3047MG**  Proneural  Passage 9 | 195 | 67 days | Intermediate |
|  | 196 | 67 days | Large |
|  | 197 | 70 days | Large |
|  | 198 | 59 days | Intermediate |
|  | 199 | 67 days | Large |
| **U3054MG**  Mesenchymal  Passage 14 | 215 | 71 days | Small |
|  | 216 | 70 days | Small |
|  | 217 | Died during surgery | NA |
|  | 218 | 70 days | Small |
|  | 219 | 58 days | Small |
| **U3065MG**  Mesenchymal  Passage 12 | 210 | 134 days | Intermediate |
|  | 211 | 58 days | Small |
|  | 212 | Died during surgery | NA |
|  | 213 | 92 days | Intermediate |
|  | 214 | 76 days | Small |
| **U3082MG**  Proneural  Passage 9 | 200 | 67 days | Intermediate |
|  | 201 | 58 days | Intermediate |
|  | 202 | 67 days | Intermediate |
|  | 203 | 67 days | Intermediate |
|  | 204 | 132 days | Large |

**Supplementary Table 2**

| **Cell Line** | **Th/HTh** | **CC** | **Cx** |
| --- | --- | --- | --- |
| U3009MG ** | 7.29% (37/507) | 5.99% (36/601) | 13.59% (39/287) |
| U3013MG ** | 15.50% (40/258) | 14.40% (56/389) | 94.66 (284/300) |
| U3017MG ** | 24.61% (63/256) | 14.04% (42/299) | 35.27% (97/275) |
| U3024MG * | 12.38 (63/509) | 9.66% (46/476) | 18.12% (100/552) |
| U3047MG ** | 10.98% (55/501) | 5.03% (26/517) | 11.84% (38/321) |
| U3054MG * | 79.84% (198/248) | 66.32% (315/475) | 97.56% (40/41) |
| U3065MG * | 12.84% (62/483) | 9.15% (27/295) | 15.07% (44/292) |
| U3082MG * | 9.92% (50/504) | 4.96% (13/262) | 9.51% (25/263) |
| Percentage vessel-associated cells (number of vessel-associated glioma cells/Total number of glioma cells counted) at invasive front regions, indicated in Supplementary Fig. 2. Cells in 2-5 coronal sections (100 µm thick) from one (*) or two (**) mice were counted. | | | |


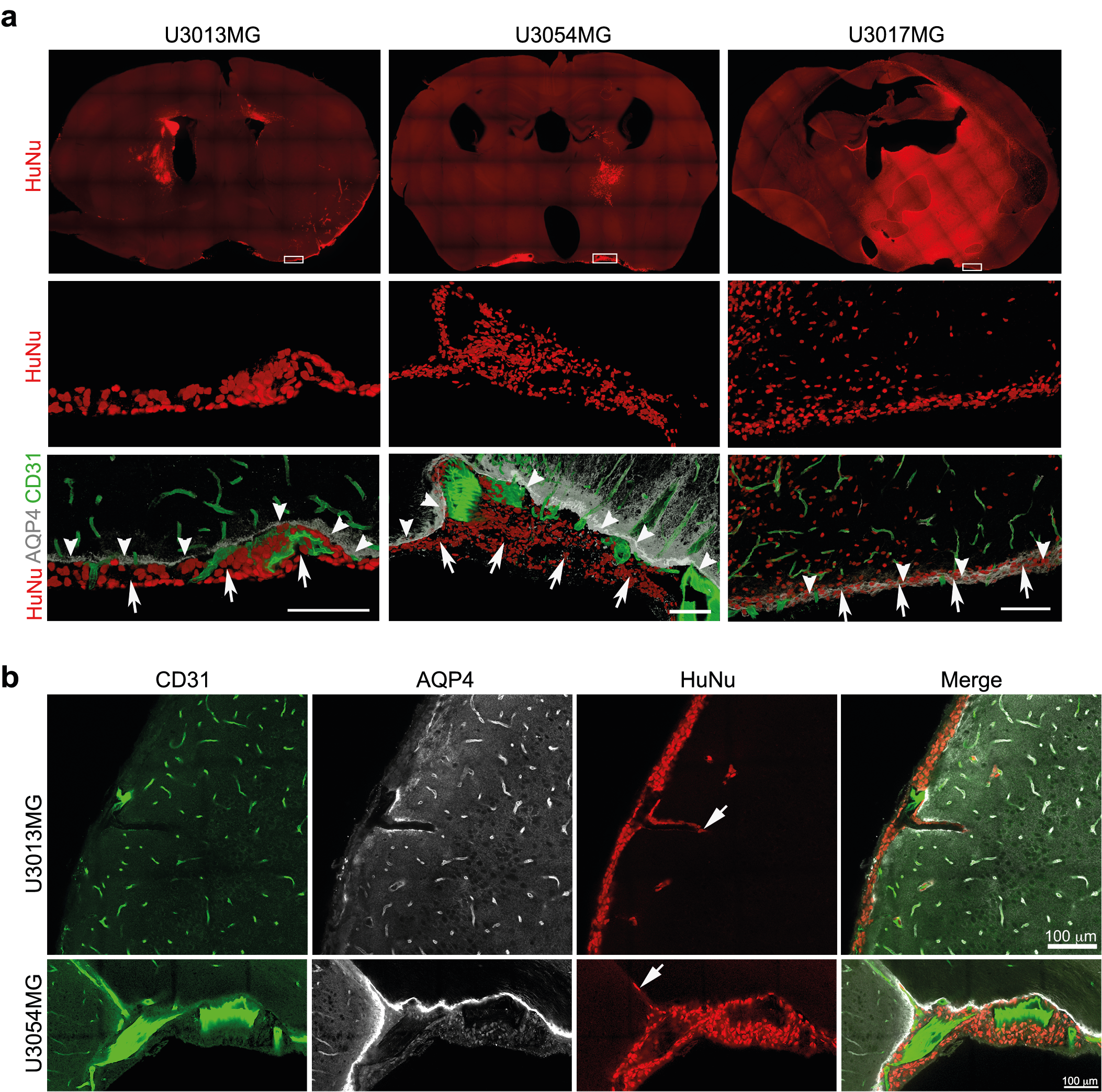


**Supplementary Fig. 1** **a-b** Tumor cell accumulation in the subarachnoid space and penetration into the brain parenchyma. Immunofluorescent staining using antibodies against HuNu, aquaporin 4 (AQP4, astrocyte end-feet) and CD31 (endothelial cells). z-stacks were taken using a Leica confocal microscope, 63X objective. Scale bars, 100 µM. **a** U3013MG and U3054MG tumor cells accumulate and move along subarachnoid space, compared to other GSC lines. Upper panel, tumor growth pattern; lower and middle panels, 3D image snapshots of limited adjacent z-stacks of the selected areas. Arrows indicate glioma cells and arrowheads indicate glia limitans superficialis. U3017MG tumor shown as an example where accumulation of tumor cells in the subarachnoid space is not observed. **b** U3013MG and U3054MG tumor cells in perivascular spaces of vessels penetrating the brain from the subarachnoid space. Arrows indicates entry of tumor cells in perivascular space from subarachnoid space. 100 µm thick coronal sections. Each image represents maximum intensity projection of few z-stacks. **See related Supplementary Movie 1.**


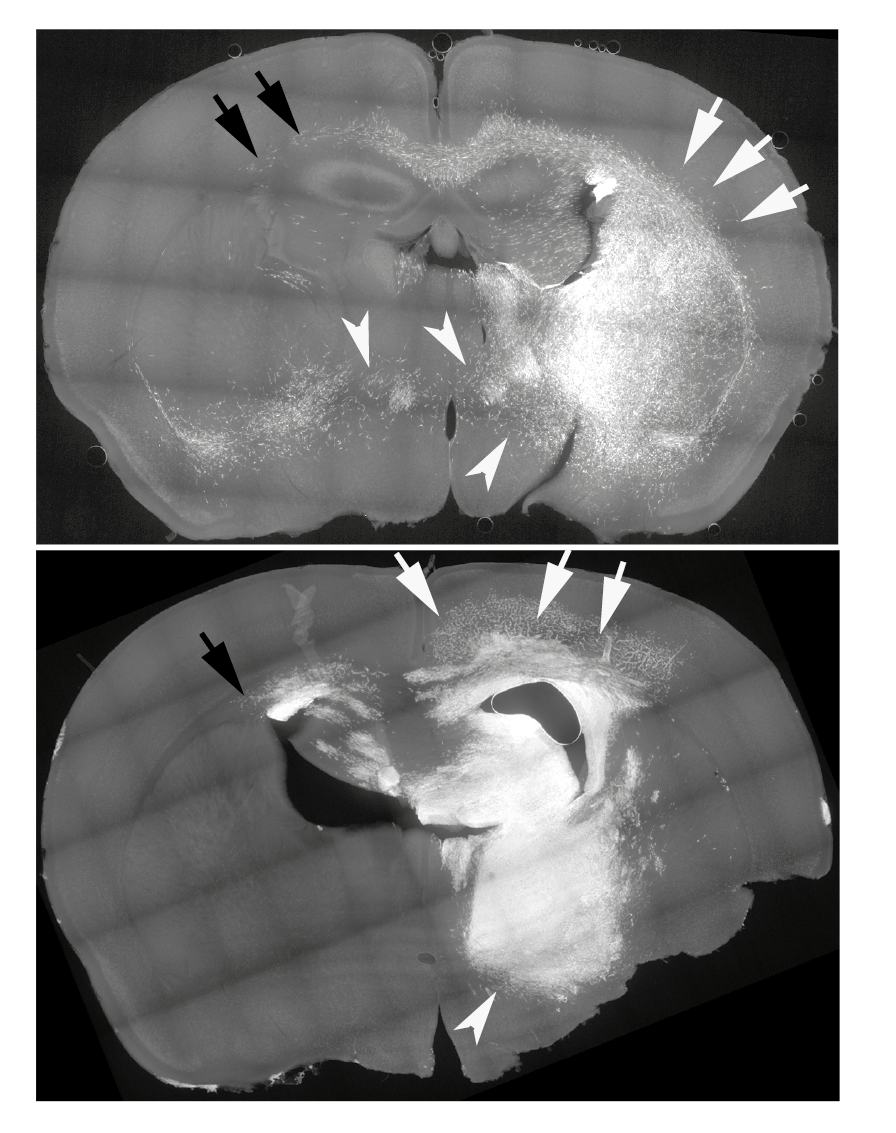


**Supplementary Fig. 2** Examples of coronal sections used for quantification of vessel-associated cells in the tumor invasive front. Three regions were analyzed: cortex, white arrows; thalamus/hypothalamus, white arrowheads; and corpus callosum, black arrows.


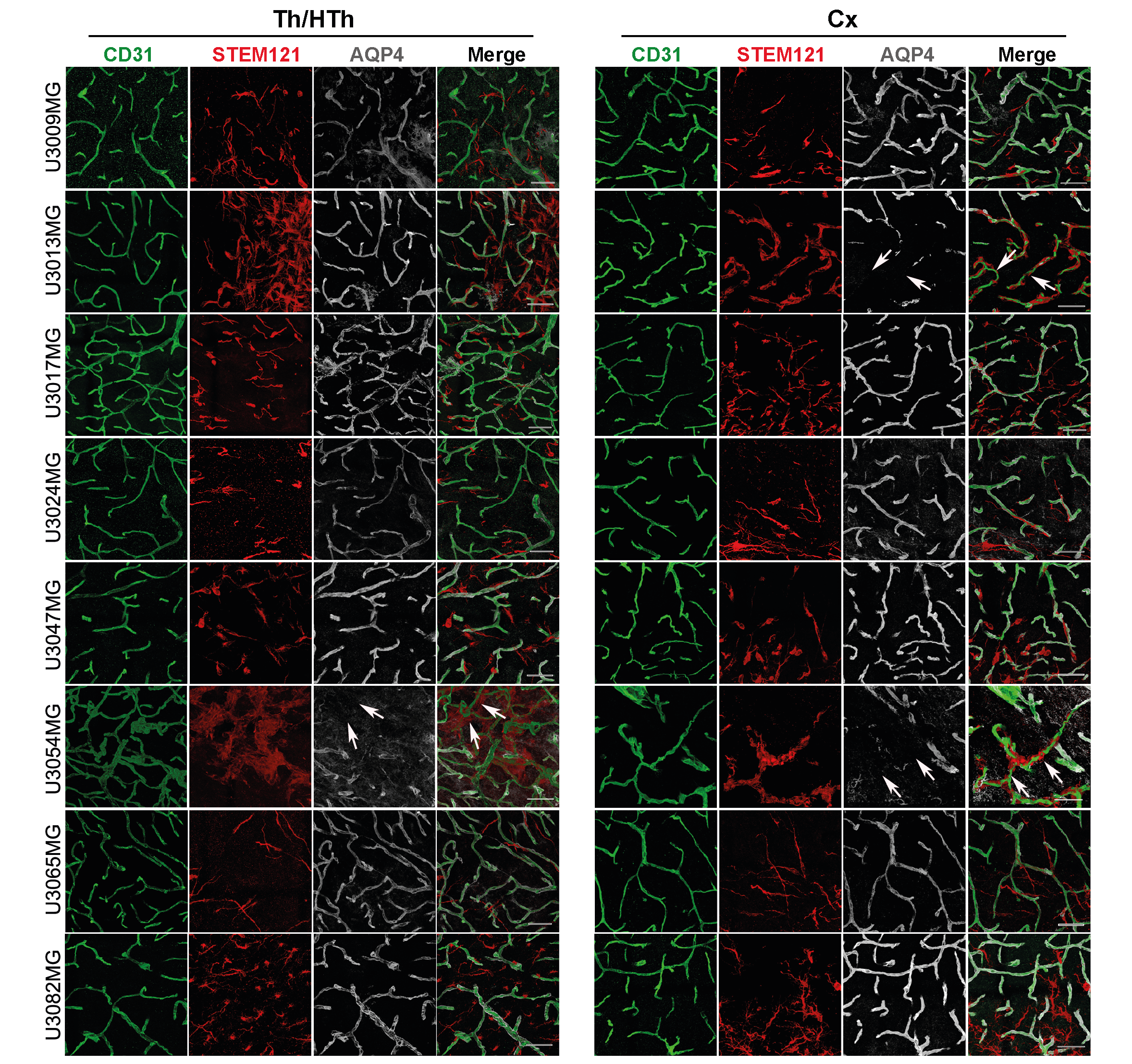
**Supplementary Fig. 3** Loss of astrocyte end-feet in regions of high-level vessel-associated glioma cells. Immunofluorescent staining of human glioma cells (STEM121, red), astrocyte end-feet (AQP4, white), and blood vessels (CD31, green) on coronal sections. z-stacks were taken by Leica confocal microscope using 63x objective in tile scan mode. Each image represents a snapshot of a 3D image. Arrow indicates the glioma cell occupied region with loss of astrocyte end-feet. Cx, cortex; Th/HTh, thalamus/hypothalamus. Scale bar, 50 µm.

**
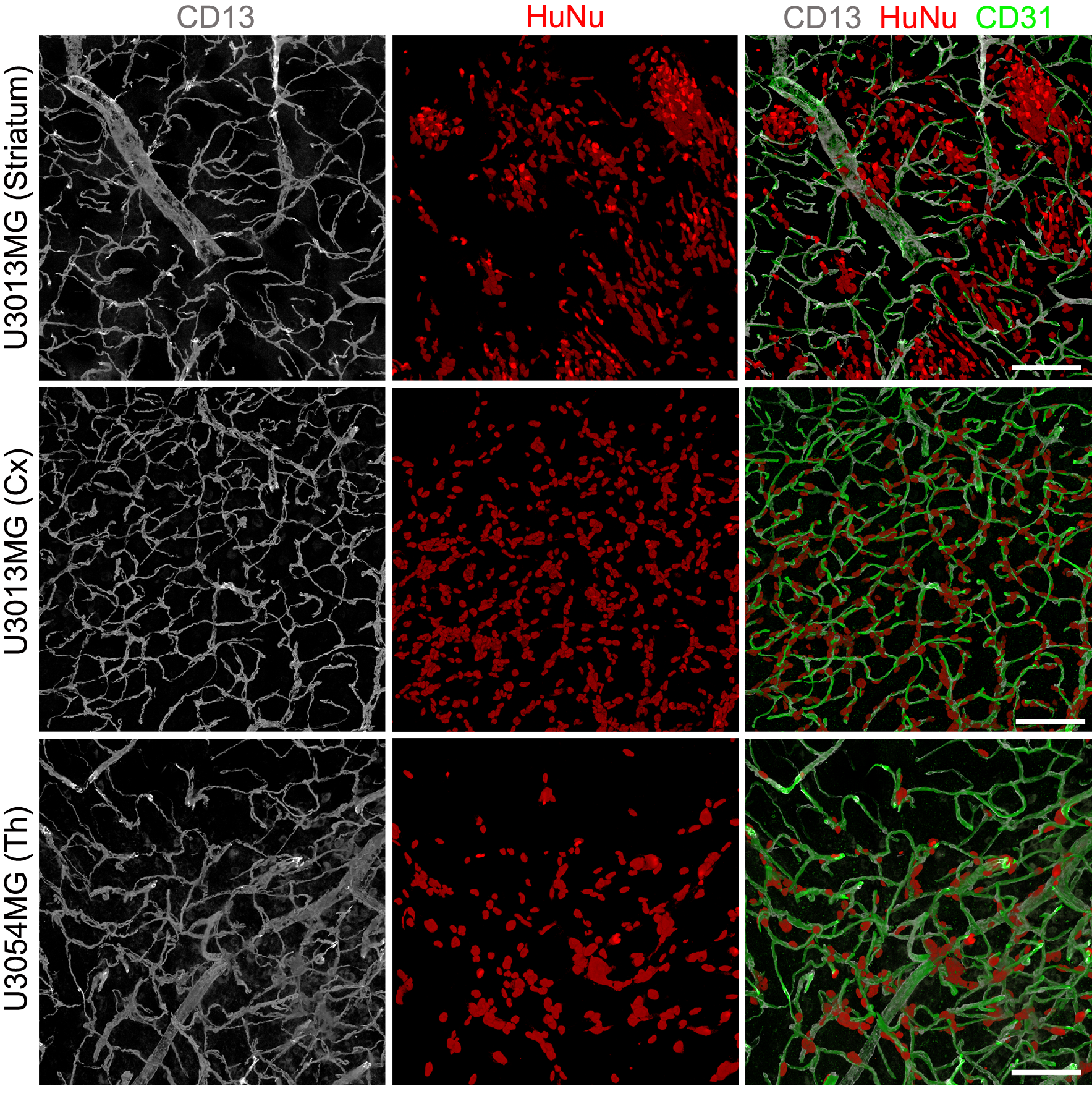
 Supplementary Fig. 4** No detectable loss of pericytes from the vessels occupied by migrating glioma cells. Coronal sections were stained with HuNu (red, human glioma cells), CD13 (white, pericytes), and CD31 (green, blood vessels) antibodies. z-stacks were taken by Leica confocal microscope using 63x objective in tile scan mode. Each image represents a snapshot of a 3D image. Cortex (Cx), Thalamus (Th). Scale bar, 100 µm.

**
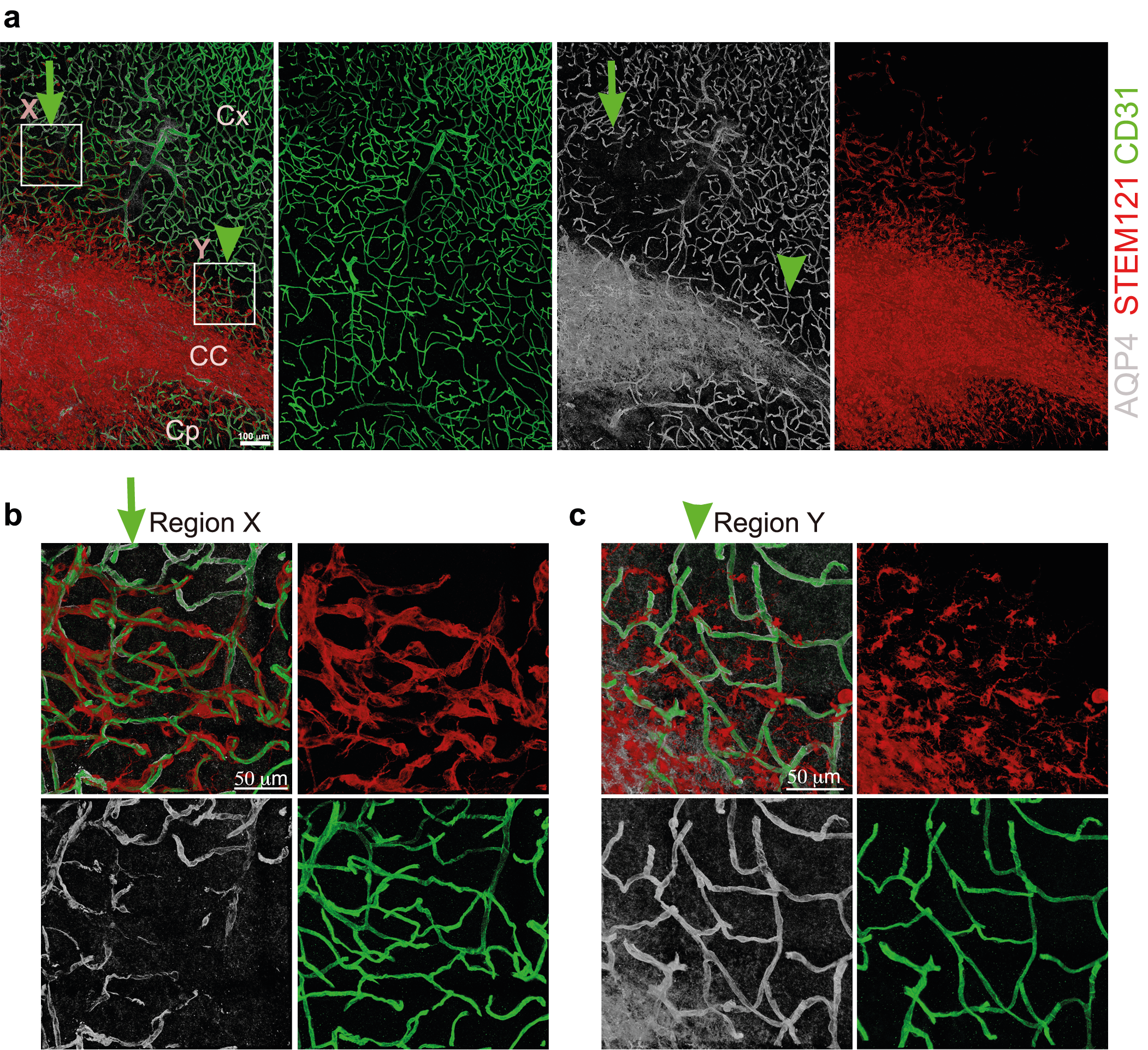
**

**Supplementary Fig. 5** U3013MG tumor cells localized in the cortex in close proximity to the corpus callosum do not displace astrocyte end-feet from the vessels. **a** Coronal sections (100 µm) were stained with STEM121 (red, human glioma cells), anti-AQP4 (white, astrocyte end-feet), and anti-CD31 (green, blood vessels) antibodies and z-stacks were taken by Leica confocal microscope using tile scan function. Boxes indicates glioma cells possibly moving from corpus callosum into cortex near the lateral ventricle (Box X) or near the tumor invasive front at corpus callosum (Box Y). **b-c** Zoom of Box X and Box Y, respectively. Each image represents a snapshot of a 3D image of selected z-stacks. Abbreviations: CC (corpus callosum), Cx (cortex), Cp (Caudoputamen).


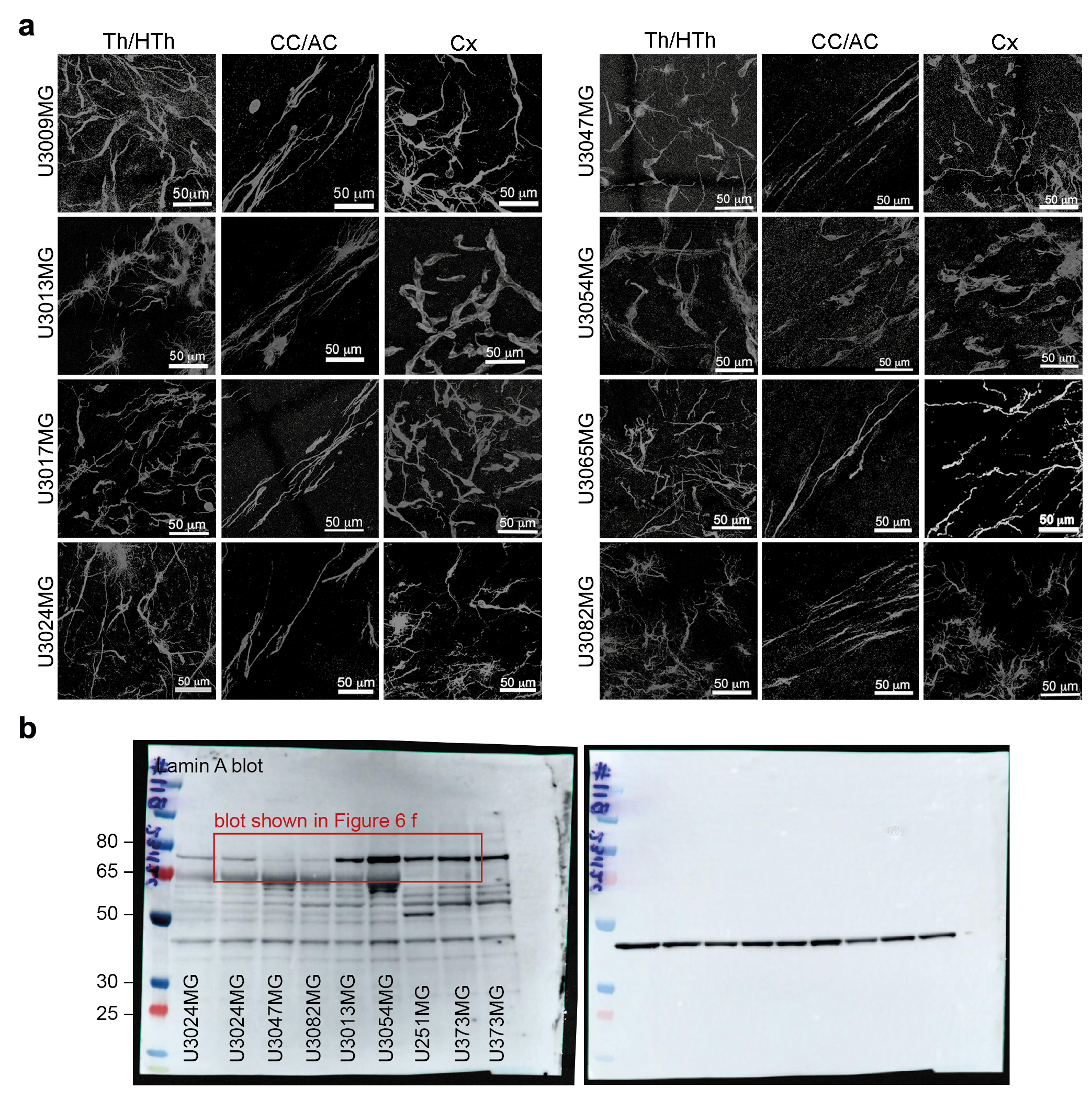


**Supplementary Fig. 6**

**a** Morphology of tumor cells in different brain regions. Coronal sections were stained with STEM121 antibody to visualize human glioma cell morphology. z-stacks were taken by Leica confocal microscope using 63X objective in tile scan mode. Each image represents a snapshot of a 3D image. Scale bar, 50 µm. Corpus callosum, CC; anterior commissure, AC; cortex, Cx; thalamus/hypothalamus, Th/HTh.

**b** Full-length blots (left, lamin A, 74 kDa; right, b-actin, 42 kDa) of cropped western blots shown in Fig. 6f. Framed lanes indicate samples used in this study.

**Supplementary Movie 1**: U3013MG tumor cells in perivascular spaces of vessels penetrating the brain parenchyma from the subarachnoid space. Immunofluorescent staining of 100 µm thick coronal sections using HuNu, aquaporin 4 (AQP4, astrocyte end-feet) and CD31 (endothelial cells) antibodies. z-stacks were taken using a Leica confocal microscope in tile scan mode, 63X objective. Stitched images from one surface to another surface of the coronal section are played as a movie. **Related to Supplementary Fig. 1b**.

**Supplementary Movie 2-25:** Association of human glioma cells (STEM121, red) with vasculature (CD31, green) at invasive fronts in corpus callosum (CC), cerebral cortex (Cx) and thalamus/hypothalamus (Th/HTh) in tumors from all eight cell lines. **Related to Fig. 2b-c.**

**Supplementary Movie 26**: Nuclear plasticity and fragmentation (micronuclei) in tumor cells migrating in the corpus callosum. 100 µm thick coronal sections were stained with HuNu (red) antibody to visualize human U3065MG glioma cells. z-stacks were taken by a Leica confocal microscope using 63x objective.  The movie represents a rotating 3D image at the x-axis. **Related to Fig. 6c.**
